# Research Progress on Gene Regulation of Plant Floral Organogenesis

**DOI:** 10.3390/genes16010079

**Published:** 2025-01-12

**Authors:** Lixia Zhou, Amjad Iqbal, Mengdi Yang, Yaodong Yang

**Affiliations:** 1 National Key Laboratory for Tropical Crop Breeding, Chinese Academy of Tropical Agricultural Sciences, Haikou 571101, China; yyang@catas.cn; 2 Hainan Key Laboratory of Tropical Oil Crops Biology, Coconut Research Institute, Chinese Academy of Tropical Agricultural Sciences, Wenchang 571339, China; amjadiqbal@awkum.edu.pk; 3 Department of Food Science & Technology, Abdul Wali Khan University, Mardan 23200, Pakistan; 4 Qionghai Tropical Crops Service Center, Qionghai 571400, China; 13137360232@163.com

**Keywords:** floral organ, gene regulation, MADS box, AP2/ERF, NAP

## Abstract

Flowers, serving as the reproductive structures of angiosperms, perform an integral role in plant biology and are fundamental to understanding plant evolution and taxonomy. The growth and organogenesis of flowers are driven by numerous factors, such as external environmental conditions and internal physiological processes, resulting in diverse traits across species or even within the same species. Among these factors, genes play a central role, governing the entire developmental process. The regulation of floral genesis by these genes has become a significant focus of research. In the AE model of floral development, the five structural whorls (calyx, corolla, stamens, pistils, and ovules) are controlled by five groups of genes: A, B, C, D, and E. These genes interact to give rise to a complex control system that governs the floral organsgenesis. The activation or suppression of specific gene categories results in structural modifications to floral organs, with variations observed across different species. The present article examines the regulatory roles of key genes, including genes within the MADS-box and AP2/ERF gene clusters, such as *AP1*, *AP2*, *AP3*, *AG*, *STK*, *SHP*, *SEP*, *PI*, and *AGL6*, as well as other genes, like *NAP*, *SPL*, *TGA*, *PAN*, and *WOX*, in shaping floral organ genesis. In addition, it analyzes the molecular-level effects of these genes on floral organ formation. The findings offer a deeper understanding of the genetic governance of floral organ genesis across plant species.

## 1. Introduction

The switch from vegetative to reproductive development signifies a major physiological transition during the growth phases of higher plants [1]. Flowering is a very important part of the ontogeny of higher plants. The differentiation of flowers begins with reproductive growth. During this process, the vegetative meristem turns into the inflorescence meristem, which then undergoes differentiation [2,3]. Subsequently, the inflorescence meristem differentiates the floral meristem, triggering the formation of floral organ primordia, which then develop into floral organs [4]. The transition from vegetative growth to reproductive development is a crucial stage in the plant life cycle. This process is precisely regulated by multiple genes to ensure that plants complete the reproductive process under appropriate temporal and environmental conditions, thus guaranteeing the reproduction of the species [5].

As a unique reproductive organ of seed plants, flowers play an important role in plants and determine whether plants can form fruits and seeds. A fully developed flower consists of five key structural components: the calyx, corolla, stamen, pistil, and ovule [6]. However, some plants such as *Eucommia ulmoides* and *Salix babylonica* only have stamens or pistils in their flowers [7,8]. Floral organ development is an extremely complex process, one which is affected by both internal physiology and external environmental factors [9]. Following the cloning of the first flower development-related gene, MADS box, in *Arabidopsis thaliana*, the genetic regulation of floral organogenesis has increasingly become a focal point of research. The entire floral organ genesis process is modulated as a consequence of the interaction of several genes. Beyond the primary genes in the AE model of floral organogenesis, several additional auxiliary genes contribute to a large and intricate regulatory network that orchestrates the process of floral organogenesis [10,11]. This paper summarizes the regulatory roles of several genes, such as *Apetala1* (*AP1*), *Apetala2* (*AP2*), *Apetala3* (*AP3*), *Agamous* (*AG*), *Agamous-like6* (*AGL6*), Pistillata (*PI*), Sepallata (*SEP*), *NAC-like, Activated by AP3/PI* (*NAP*), *Squamosa-promoter binding protein-like* (*SPL*), *TGACG motif-binding factor* (*TGA*), *Perianthia* (*PAN*), *Wuschel-related Homeobox* (*WOX*), *Seedstick-like* (*STK*), and *Shatterproof* (*SHP*), in the plant floral organogenesis. This provides a foundation for more detailed study on the genetic regulation of flowering across various plant species.

## 2. Models of Plant Floral Organogenesis

The concept of floral organ genesis models was initially introduced in the early 1990s by Coen et al. [12] and Meyerowitz [13] via their investigations into *Arabidopsis* and *Antirrhinum*. Their work led to the formulation of the ABC model of floral organogenesis, which offered valuable insights into the structure, developmental processes, and regulatory mechanisms underlying floral development [14]. Later, Colombo et al. identified D-class genes that control ovule development in petunia plants, leading to the proposal of the ABCD model [15]. In 2001, Theissen et al. further expanded on this research by introducing the ABCDE (AE) model. This model has since become the current and widely used model in floral organogenesis studies, incorporating new insights and findings to provide a more comprehensive understanding of the genetic control of floral organ development [16].

According to the AE model, the development of the calyx and corolla, which make up the first two tiers of floral organs, is mainly regulated by A-class genes. In a flower, the floral organs are arranged in concentric circles called whorls, and the calyx and corolla are located in the outer whorls.

**Figure 1:**
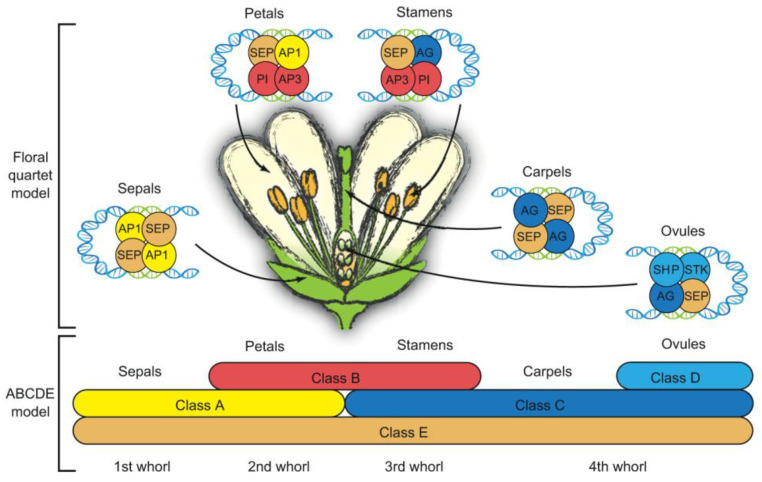
ABCDE model and tetramerization model of floral morphogenesis in plants [17].

B-class genes are responsible for controlling the development of the corolla and stamen, which are the second and third layers of floral organs. In some plants, such as tulips and certain orchid species, B-class genes can also have an influence on the calyx. This shows the complex regulatory role of these genes in different plant species. The third and fourth layers of floral organs, including the stamen, carpel, and ovule, are regulated by C-class genes. However, in some plants, like maize and rice, C-class genes may only affect the stamen and carpel. Ovule development, on the other hand, is driven by D-class genes [17]. E-class genes play a crucial role in governing the formation of floral organs in all whorls. They interact with A-, B-, and C-class genes to form complexes, a mechanism known as the “tetramer model”. In this model, the E-class genes act as a kind of glue, binding with other classes of genes to ensure the proper development and arrangement of floral organs (Figure 1) [17].

**Figure 2:**
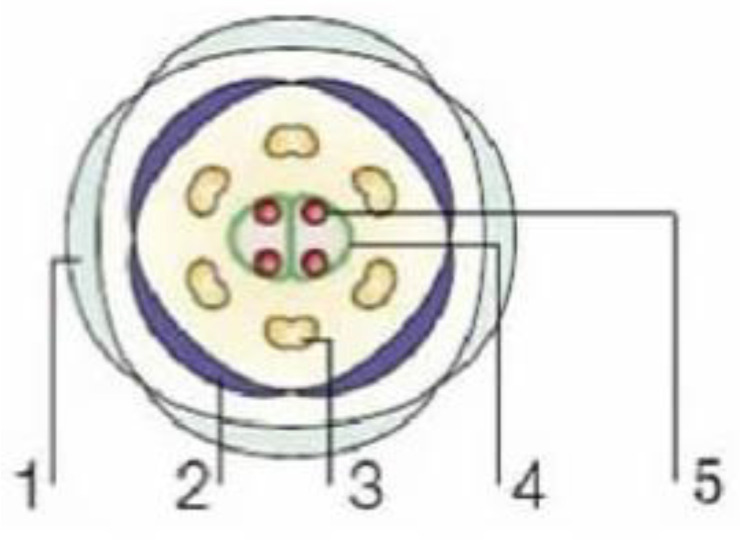
The five-whorl structure of flowers. 1–5: calyx, petal, stamen, carpel, ovule.

The AE model effectively explains floral morphogenesis in most dicotyledonous plants. Additionally, the activity patterns of homologous genes in monocotyledonous plants provide evidence for the model's relevance to monocot grasses. In these grasses, the lemma and palea are analogous to the calyx and petals in dicotyledonous plants. However, the AE model exhibits some variations in non-grass monocotyledonous plants. In the AE model, the five gene classes work together to regulate the five whorls of floral structures (Figure 2). Mutations in any of these gene classes result in altered floral morphologies (Table 1).

## 3. The Roles of the AE Floral Organ Model in Flower Development

### 3.1. The Functional Diversity of AP1 and AP2 Genes in Floral Organ Genesis

*AP1* and *AP2* are the key genes in class A. *AP1*, a member of the MADS-box family, plays crucial roles in floral development. The MADS-box family comprises a large group of genes that are widely involved in various aspects of plant growth and development, especially in the regulation of floral organ identity. *AP1* serves as both a central gene in the floral meristem, which is a region of undifferentiated cells that give rise to the different parts of the flower, and a defining gene for the morphological traits of floral tissues. During floral organogenesis, the interaction between *AP1* and the *AP2* gene is of great significance. This interaction can lead to the specialization of sepals and petals. For example, it may determine the specific shape, size, and color patterns of these floral organs. At the same time, this interaction can also activate class-B genes, which are involved in the development of other floral structures. The protein encoded by *AP2* functions as a transcriptional activator. It regulates gene transcription by connecting to particular DNA sequences. For instance, it may bind to specific promoter regions of target genes, where it can recruit other transcriptional machinery components to initiate or enhance gene expression. This precise regulation is essential for the proper development and functioning of floral organs [18]. The *AP2* gene encodes proteins belonging to the AP2/EREBP transcription factor family and is expressed in both the vegetal and reproductive tissues of plants [15]. In reproductive tissues, it performs a central role in the formation of sepals and petals, while also suppressing the activity of class C genes. The flowers of plants with miR172 target site mutations show phenotypes with enlarged meristems and excessive stamens. A deficiency in *AP2* function results in stamens taking the place of petals, whereas its abnormal expression can induce the formation of double petals in flowers. In transgenic *A. thaliana*, the enhanced activity of *RcAP2* leads to the conversion of stamens into petals, resulting in an increased petal count, whereas silencing *RcAP2* decreases the number of petals [19]. The misexpression of *AP1* and its related genes induces earlier flowering in *Arabidopsis*. However, the roles and expression patterns of *AP1* and *AP2* genes differ across various species (Table 2).

### 3.2. Molecular Regulation of Petals and Stamens by AP3 and PI in Flower Morphogenesis

*AP3* and *PI* are B-class genes in the MADS-box family that are crucial modulators of floral organogenesis, particularly in the formation of stamens and petals. The promoter of the *AP3* gene is organ-specific, playing a significant part in the development of petal and stamen cells. The *PI* gene, through its transcription factor binding to specific DNA regions, regulates the morphogenesis of petals and stamens. Moreover, *PI* is expressed in the petals and stamens of *Arabidopsis*, where it performs a pivotal function in defining their identities. The roles of *AP3* and *PI* in floral organogenesis have been established and corroborated in other species (Table 3).

*AG* is a C-class gene in the MIKC^c^-type *MADS-box* family, governing stamen and carpel differentiation and regulating floral meristem termination [43]. Sage-Ono et al. also used gene integration techniques in morning glory to successfully induce double-flowered morning glory [44]. In recent studies, AG’s contribution to floral morphogenesis has been proven in other plant species (Table 4).

In the AE model of flower development, the regulation of ovule formation is primarily governed by class-D genes, specifically *STK* (SHATTERPROOF-like) and *SHP1/2* (*SHATTERPROOF1/2*). The STK gene belongs to the MADS-box gene family and plays a crucial role in regulating the normal development of carpels and ovules. The normal expression of the STK gene is essential for the initiation and differentiation of carpel primordia. It participates in processes such as the fusion of carpel margins, ensuring that carpels can develop normally to form a closed ovary structure and providing a suitable environment for the development of ovules. Meanwhile, the STK gene also affects the number and distribution of ovules. Changes in its expression level may lead to abnormal ovule development, such as decreases in the number of ovules or uneven distributions. The STK gene also plays an important regulatory role in the formation of the overall morphology of the pistil. It interacts with other genes to jointly regulate processes such as cell division, differentiation, and elongation, thereby determining the size, shape, and structure of the pistil. Ovules are important structures for the development of female gametophytes and produce seeds after fertilization [50]. The *STK* gene in *A. thaliana* is predominantly expressed in the ovary, where it performs a central task in controlling the development of both ovules and seeds. The *stk*, *shp1*, *shp2*, and *stkshp1shp2* triple mutants all cause normally developing ovules to transform into structures resembling carpels or leaves [50]. The *STK* homologous gene *PrseSTK* in double-flowered *Prunus serrulata* ‘Fugenzo’ is active in stamens, sepals, and pistils. Its misexpression in sepals leads to the formation of ectopic ovaries on the calyx tube of double-flowered cherry blossoms, and thus it is involved in regulating the morphological differences between single-petaled and double-petaled cherry blossoms [51]. In *Phalaenopsis*, the *phSTK* gene showed predominant expression in the column, ovary, and ovules, with the maximum level of expression observed in the ovules. Its expression intensifies progressively as the ovary develops. In the staminode petaloid mutant, the *PhSTK* gene showed a notable reduction in expression in the column, while its expression in the ovary was considerably elevated. Conversely, in the lateral-petal-masculinized mutant, *PhSTK* expression was significantly higher in both the column and the ovary [52]. The *STK* homologous gene *EpMADS23* in *Oncidium* exhibited substantially higher expression in the gynostemium compared to other floral organ tissues [53]. The *CygoSTK* gene in *Cymbidium goeringii* is predominantly expressed in the lip, pollinia, gynostemium, and ovary, with particularly high expression levels in the ovary. This indicates its potential role as a key regulator of ovary development in this species [54]. The mutation of the *ZmSTK2* gene significantly reduces the germination rate of maize pollen. At the S11 stage, mutant pollen displays a reduced abundance of starch grains and a marked increase in lipid body accumulation compared to wild-type pollen [55].

### 3.3. Involvement of SEP Genes in Floral Organ Formation and Flowering Regulation

*SEP* (*SEPALLATA*) is a member of the E-class genes in the AE model, playing a remarkable role in regulating the development of sepals, petals, stamens, and pistils. In *A. thaliana*, the *SEP* genes (*SEP1/2/3/4*) collaborated with the A-, B-, and C-class genes to control the development of carpels, stamens, and petals. The protein complex generated through the concurrent expression of *SEP* and the A-, B-, and C-class genes has the ability to convert vegetative leaves into floral tissues, thus reducing the vegetative growth phase and further affecting the flowering time [56]. Extensive research by numerous scientists has led to the cloning of *SEP* genes in various species, with their functions being confirmed through subsequent studies (Table 5).

### 3.4. Contribution of Other Genes in the Modulation of Floral Organogenesis

Apart from the genes associated with the AE model of flower morphogenesis, genes like *AGL6*, *NAP*, *SPL*, *TGA*, *PAN*, and *WOX* also contribute to various aspects of flower organogenesis [65,66]. The *AGL6* gene, a member of the MADS-box gene family, performs a role in regulating the differentiation of floral meristems, as well as in the development of floral organs and ovules [67]. When homologs of *AGL6* from various species were transferred into *A. thaliana*, they induced early flowering. For instance, the *Oncidium Gower Ramsey AGL6* homolog, *OMADS1*, and the *Magnolia wufengensis AGL6* gene, *MawuAGL6*, both promoted early flowering in *A. thaliana* [68]. The *SlAGL6* gene demonstrated a substantial role in the development of sepals, petals, and carpels in tomatoes. The silencing of *SlAGL6* may cause the abnormal fusion of sepals in plants, and the petals may become smaller and turn light green. In tomato plants that were genetically engineered with the *SlAGL6* gene, the expression of genes associated with sepal development, such as *Macrocalyx* (MC) and *AP2a/c*, was significantly reduced. Additionally, the expression levels of C-, D-, and E-class genes within the AE model were also altered [69].

A study indicated that the interaction between B- and E-class genes, along with AGL6, played a central role in the evolutionary development of orchid flowers [70]. The B-class genes and AGL6 proteins were assembled into two complexes, SP (OAP3-1/OAGL6-1/OPI) and L (OAP3-2/OAGL6-2/OPI), which worked together to regulate the development of orchid lips, sepals, and petals. Specifically, the SP complex was responsible for the formation of sepals and petals, while the L complex governed the development of the lip [71]. *AGL6* and Floral Binding Protein 2 (*FBP2*) have been identified as crucial regulators of petal development in petunia plants, possessing overlapping functions. In the *fbp2* mutant, the edges and the back along the main veins of the petals were transformed into green sepal-like structures, and this phenomenon was more obvious in the double mutant *fbp2agl6*. The corolla became smaller and the petals turned green. The *phagl6fbp2* double mutant displayed structures resembling sepals or petals at the tips of the anthers, and occasionally, structures similar to a stigma were also present [72].

A study investigating wild-type *A. thaliana*, the *35S::antiNAP* suppression line, and the *35S::NAP* overexpression line revealed notable differences in the petals and stamens across the three lines [73]. In the overexpression line, both the petals and stamens were shorter compared to the wild type, and the plants produced sterile flowers. In contrast, the suppression line exhibited shorter stamens than the wild type, with anthers that failed to dehisce. The *AtSPL3* gene, located in the flower primordium and floral meristem of *A. thaliana*, can influence the transformation of floral organs by regulating the *Fruitfull* (*FUL*), *Leafy* (*LFY*), and *AP1* genes, thereby promoting earlier flowering in plants [74]. *AtSPL4* and *AtSPL5* primarily regulate the genes *FUL* and *Suppressor of Overexpression of CO1* (*SOC1*), playing a role in controlling the floral cycle. The overexpression of *AtSPL3/4/5/9/10* accelerates the transition of *A. thaliana* into the reproductive growth stage [75], while the overexpression of *AtSPL9* significantly reduces flowering time [76].

*PAN* is a distinctive gene within the *TGA* family. A mutation in this gene leads to a phenotype in *A. thaliana* characterized by the presence of five sepals and five petals [77]. *PAN* interacted with *Blade-on-petiole1/2* (*BOP1/2*), and the *bop1/2* double mutant exhibited a phenotype with five sepals. However, the triple mutant *bop1bop2pan* did not show more pronounced abnormalities. *PAN* is capable of interacting with *ROXY1*. In the *roxy1-2* mutant, the average petal count was 2.5, whereas the double mutant *roxy1-2pan* displayed a phenotype with five petals. Additionally, *TGA9* and *TGA10* was found to have role in the regulation of flower development [78]. *TGA9* and *TGA10* were noticed to be involved in flower development, sharing both sequence similarities and comparable expression patterns. Both the *tga9/10* and *roxy1/2* double mutants exhibited defects in anther development. Furthermore, *TGA9* and *TGA10* can directly interact with *ROXY1* and *ROXY2* [79]. Knocking down *TGA2.1* in tobacco resulted in the transformation of tobacco stamens into petal-like structures [80]. *AtWOX13* and *AtWOX14* were involved in the flowering process of *A. thaliana*. *AtWOX13* was mainly expressed in the vascular tissue, stigma, and pistil during flower stages 13/14. On the other hand, the *wox14* mutant showed poorly developed stamens after flowering, which disrupted pollination and fertilization, leading to the abortion of ovules [81]. A study revealed that *OsWOX13* only expressed during the early development of male reproductive organs and in the female organs at maturity, with the overexpression of *OsWOX13* causing plants to flower 7-10 days earlier [82]. The tomato *WOX* gene *Uniflora* (*UF*) influenced flower development, with the *uf* mutant exhibiting significant defects in petals, carpels, stamens, and lateral growth [83]. In *A. thaliana*, plants overexpressing the *TaWuschel* (*WUS*) flower earlier than the wild type exhibited a higher number of flower buds, and showed a considerable increase in petal count, while the number of pistils and stamens remained unaffected [84]. Advancements in molecular biology have led to the discovery and reporting of numerous genes involved in flower development, with researchers progressively unraveling the mechanisms through which these genes regulate the formation of floral organs across different plant species.

## 4. Molecular Regulation of Floral Organogenesis and Its Implications for Ornamental and Economic Crops

### 4.1. Implications for Ornamental and Economic Crops

Flowers serve as the reproductive organs of plants, with the morphology of floral organs reflecting both the diversity of plant species and their adaptation to environmental conditions [85]. The floral organs of different plants have different advantages in production practice, such as enhancing ornamental value, strengthening stress resistance, and increasing yield per unit area [86]. The rapid progress in molecular biology and the growing focus on multi-omics fields, like transcriptomics, genomics, metabolomics, and proteomics, have made the study of genes regulating floral organogenesis a prominent research area, leading to the discovery and reporting of numerous genes involved in this process [87].

For the vast majority of flowering plants, such as *Magnoliaceae* [88], *Ericaceae* [89], *Oleaceae* [90], *Orchidaceae* [91], and *Rosaceae* [92], flowers are important for ornamental purposes. Certainly, the number, size, color, shape of petals, and flowering time all directly influence the ornamental value of flowers. Studying the morphological structure and flowering time of various flowering plants through gene regulation and the breeding of new varieties with higher ornamental value according to certain requirements is an important approach in the research of ornamental plants. Secondly, studies on the AE model of floral organogenesis have demonstrated that the deletion or altered expression of various genes can result in the transformation of the five whorl structures of floral organs [93]. Madrigal et al. conducted phylogenetic analyses of the COL/COL4, FD, FLC/FUL, and SOC1 gene families. They found that, due to gene family duplication events specific to Orchidaceae plants, the number of homologous genes belonging to the *COL4* and *FUL* gene families in Orchidaceae plants increased compared with other monocotyledonous plants (including grasses). In addition, local duplication events of the *COL*, *FD*, and *SOC1* gene families occurred less frequently, indicating that some important functions in key signaling factors are retained under strong purifying selection conditions [89]. The structure of floral organs can be changed by means of gene regulation, and the number of petals can be increased to enhance the ornamental value of flowers [94]. Flowers, as the distinct reproductive organs of seed plants, are pivotal for pollination, fertilization, and the execution of reproductive processes. In many economically important crops, including food crops and fruit trees, the yield and quality of fruits and seeds serve as key indicators of their value [95]. The yield for a given year is influenced by both the number of flowers and the timing of flowering. Once the flower count is assured, the floral structure becomes a critical factor in determining whether the plant can successfully produce fruits. Modifying the expression of flowering-related genes can enhance the number of stamens, thereby increasing pollen production and improving the likelihood of successful pollination and fertilization. Gene regulation can be utilized to develop stronger pistils and ovules or to enhance their resilience under challenging conditions. In addition, with the increasing frequency of extreme weather events, the adjustment of flowering times to avoid adverse conditions or improve stress resistance represents a critical area for future research in economically important crops [96]. In ornamental plants, ethylene is an important plant hormone that is closely related to the senescence and wilting processes of flowers. Taking carnations as an example, researchers have introduced antisense genes, encoding key enzymes in the ethylene synthesis and signal transduction pathway into carnation plants through genetic engineering techniques. These antisense genes can inhibit the synthesis or signal transduction of ethylene, thereby delaying the senescence process of flowers.

### 4.2. Molecular Regulation of Floral Organogenesis

Beyond the ABCDE-class genes, numerous other flowering-related genes interact within a complex network to collectively regulate the flowering process in plants. Within this network, the *AP1* gene serves as a critical regulatory hub [97]. The *LFY* gene promotes the expression of the *AP1* gene via a hormone-mediated regulatory pathway. Positioned downstream of *LFY*, the *AP1* gene also exerts a positive influence on LFY expression, creating a mutually reinforcing regulatory relationship [98]. In mutants of the *LFY* gene, *AP1* gene activity is not entirely lost, suggesting that the expression of the *AP1* gene is influenced by multiple regulatory factors. The *FT* and *LFY* genes independently regulate the *AP1* gene. The *FT* protein, once transported to the apical meristem, activates the *FD* gene, and together, the *FT* and *FD* protein complexes work to stimulate *AP1* gene expression [99]. The *SPL3* gene promotes early flowering by regulating *AP1* gene expression, while the *CO* gene indirectly influences *AP1* expression through the *GR* induction system. Conversely, *TFL1*, *TFL2*, and *FWA* negatively regulate *AP1* expression, although *AP1* also suppresses *TFL1* expression. Furthermore, genes like *FCA* and *AG* interact with *AP1*, highlighting its involvement in a complex regulatory network [99].

Genes of types A, B, C, D, and E have been widely studied and reported. However, for many genes, the research has only stayed at the levels of gene cloning, sequence analysis, and expression. The functional research on relevant genes is relatively scarce, or is not in-depth and systematic enough. More in-depth research on the regulatory functions of individual flower development genes can be carried out through various research methods. However, the development process of plant reproductive organs is extremely complex and involves many different types of regulatory factors. Therefore, the research on plant reproductive development systems biology will deepen the understanding of the molecular regulatory system of plant reproductive development. These research works will require the comprehensive application of large-scale transcriptome sequencing technology, proteomics technology, and bioinformatics technology.

## 5. Limitations of Gene Research Methods

### 5.1. Limitations at the Technical Level

Although modern sequencing technologies are highly advanced, there is still a certain error rate in sequencing. For example, in next-generation sequencing (NGS), due to issues such as sequencing depth and base recognition, the misjudgment of single-nucleotide variations (SNVs) may occur [100]. Especially in low-quality regions or repetitive sequence regions, the error rate may be even higher. This may lead to incorrect results in high-precision research, such as the genetic diagnosis of rare diseases.

Different sequencing technologies have different limitations in terms of sequencing length. For example, next-generation sequencing technologies usually produce relatively short sequencing read lengths (generally around several hundred base pairs), which poses challenges for the analysis of long-fragment gene structures and the detection of genomic structural variations. Although long-read sequencing technologies (such as PacBio and Nanopore sequencing) can solve some problems related to long-fragment sequencing, their sequencing costs are relatively high, and their data accuracy is relatively low, requiring further correction and validation [101].

### 5.2. Limitations of Gene Editing Technologies

Gene editing technologies represented by CRISPR/Cas9 may have off-target effects during application. That is, the Cas9 protein may cut at non-target sites, leading to unexpected modifications of other genes in the genome. This may introduce new gene mutations, affect the normal functioning of cells, and even lead to serious consequences such as cell carcinogenesis. For example, in some clinical trials of gene therapy, off-target effects are issues that need to be closely monitored and addressed [102]. The editing efficiency of gene editing technologies may vary under different cell types, gene loci, and experimental conditions. Some gene loci may be difficult to edit effectively due to their special chromatin structures or sequence characteristics. This limits the application of gene editing technologies in certain specific gene research projects and gene therapies.

## 6. Limitations at the Sample and Data Levels

### 6.1. Problems with Sample Representativeness

In gene research, the selection of samples is often limited by various factors and may not fully represent the entire research population. There may be differences in gene frequencies, genetic backgrounds, etc., among different populations. If the research samples do not cover sufficient population diversity, the research results may be biased and difficult to generalize to other populations.

Due to various practical reasons, such as research costs and the difficulty of sample acquisition, the sample size in gene research may be relatively small. A small sample size may fail to detect some rare gene mutations or weak gene associations, leading to false negatives or false positives in the research results. For example, in the gene association studies of some complex diseases, a large number of samples are needed to accurately identify gene variations related to the diseases [103].

### 6.2. Complexity of Data Interpretation

Although genome sequencing technologies can quickly obtain a large amount of gene sequence information, the specific functions of many genes remain unclear. Even if variations in certain genes are discovered, it is difficult to determine the causal relationship between these variations and specific phenotypes or diseases. For example, in genome-wide association studies (GWAS), although a large number of gene loci related to diseases can be found, the biological functions and pathogenic mechanisms of most of these loci are still unclear. There are complex interaction networks among genes, including gene–gene interactions (such as epistatic effects) and gene–environment interactions [104,105,106]. These interactions make the interpretation of gene data more difficult. For example, gene variation may exhibit different phenotypic effects under different genetic backgrounds or environmental conditions, which requires the consideration of multiple factors in the analysis of gene data [107].

### 6.3. The Necessity of Conducting Translational Research

Translational research emphasizes application orientation, enabling researchers to pay more attention to the needs of society and the market, thus conducting research work in a more targeted manner. For example, in the agricultural field, in response to the needs of food security and environmental protection, researchers apply the achievements of basic research to the cultivation of new crop varieties with high yields, pest and disease resistance, and salt-alkali tolerance through translational research, thereby improving agricultural production efficiency and the sustainable development capacity [108].

Via close integration with practical applications, translational research can promptly screen out research projects with application prospects and avoid wasting resources on research directions that have no practical application value. Translational research usually requires the participation of researchers from multiple disciplines, promoting interdisciplinary cooperation and exchanges. Through cooperation, researchers from different disciplines can integrate their respective advantageous resources and jointly tackle complex scientific problems. The construction of translational research platforms can promote the sharing of scientific research facilities and data, improving the utilization efficiency of resources. Translational research can apply the research achievements in environmental science and health fields to environmental pollution control and health protection [3]. Through translational research, agricultural scientific and technological achievements can be applied to agricultural production and food processing fields, increasing the yield and quality of agricultural products and ensuring food safety.

## Figures and Tables

**Table 1:** ABCDE-class gene function and mutant phenotype.

Main Features	Mutant Phenotype
Determine the formation of floral meristems and the initiation of sepal and petal floral organ primordia.	Strong mutants: sepals undeveloped or transformed into leaf-like structures.
Together with *AP1*, determine the initiation of the sepal and petal two-whorl floral organ primordia.	A four-whorl floral organ structure with carpels, stamens, pistils, and carpels from the outside in.
*AP3* and *PI* jointly regulate the development of petals and stamens.	When the petals and stamens are, respectively, transformed into sepals and carpels, the strong *AP3* mutant will present an enlarged central stamen and no petal structure.
Regulate the development of stamens and carpels.	The flower turns into a five-whorl structure of sepal, petal, petal, sepal, and sepal, and in some plants, the ovule mutates into a small flower with only petals.
Regulate ovule development.	The flower transforms into a structure with a sepal, petal, stamen, and pistil, and some ovules degenerate into leaf-like or carpel-like forms.
Flower organ development co-factors, participating in the development of five-whorl flower organs.	The *sep1sep2sep3sep4* quadruple mutant causes all five-whorl structures of the flower to become leaf-like structures, while the *sep1sep2sep3* triple mutant results in the transformation of the five-whorl structures into sepals.

**Table 2:** Role of *AP1* and *AP2* gene in floral development.

Species	Function Description	Reference
*Rosa chinensis*	The *RcAP1* gene enables the transition from inflorescence meristem to floral meristem, making it flower earlier, and regulates the formation of sepals during floral organ development.	[20]
*Camellia japonica* L.	*CjAP2* is involved in the sepal petalization process, resulting in the double-petal phenomenon in camellia.	[21]
*Petunia hybrida* (Hook.) E. Vilm.	The transgenic petunia R_0_ generation plants exhibit the characteristics of early and continuous flowering.	[22]
*Malus pumila* Mill., *Cymbidium* ssp.	When the apple *MdMADS2* and orchid *OMADS1* genes are transferred into tobacco, both cause the phenomena of early flowering and changes in floral organs in tobacco.	[23]
*Glycine max* (L.) Merr.	The overexpression of the soybean *GmAP1* gene in tobacco can promote early flowering in tobacco, cause the specialization of floral organs, and affect the formation of floral meristems.	[24]
*Arabidopsis thaliana*	The overexpression of *CtMADS24* leads to the upregulation of some flower development characteristic genes, thereby shortening the flowering time, while the flowering time of the silenced lines is significantly delayed.	[25]
*Lilium longiflorum* Thumb.	The overexpression of the three genes, *LMADS5*, *LMADS6*, and *LMADS7*, causes the plants to flower earlier. Homeotic transformation produces carpelloid sepals and stamenoid petals.	[26]
*Betula platyphylla* Suk	The overexpression of the *BpAP1* gene will cause early flowering in Betula platyphylla, and also affect the expression of many flowering-related genes and the synthesis of diterpenoid compounds. The Betula platyphylla offspring inheriting the *BpAP1* gene still show early flowering and fruiting, and transgenic *BpAP1* tobacco also shows the early flowering trait.	[27]
*Prunus mume*	After the *PmAG* overexpression vector was transferred into wild-type *Arabidopsis thaliana*, the petals and stamens of the transgenic plants degenerated, and the inflorescences and pods showed abortion phenomena.	[28]
*Fragaria × ananassa* Duch.	The transgenic lines show obvious early flowering, abnormal floral organs and are unable to form seeds. Meanwhile, vegetative growth is inhibited, resulting in dwarf plants and a reduced number of rosette leaves.	[29]
*Sagittaria sagittifolia* L.	The overexpression of *SsAP2* delays the flowering time and increases the number of petals in *Sagittaria sagittifolia*.	[30]
*Picea abies*	The overexpression of *PaAPETALA2-LIKE2* (*AP2L2*) leads to an increase in the number of pistils and stamens in *Arabidopsis thaliana* and postpones the flowering time. It can determine petal characteristics in the ap1 mutant of Arabidopsis thaliana. In petunia plants, the expression signal intensity gradually decreases with the maturation of organs in the outer layers of organs such as bracts, sepals, petals, and ovary walls, showing a spatiotemporal pattern.	[31]

**Table 3:** Role of *AP3* and *PI* gene in floral development.

Species	Function Description	Reference
*Arabidopsis thaliana*	When the *Arabidopsis thaliana PI* gene *AtPI* is transferred to tobacco, the floral organs of tobacco obviously show phenomena such as a smaller corolla, shorter stamens, and abnormal fruits and ovaries.	[32]
*Catalpa bungei* C. A. Mey	When the *PI* gene *CabuPI* was transferred into *Arabidopsis thaliana*, the *Arabidopsis thaliana* with the *35S:CabuPI* gene produced normal petals and different numbers of stamens.	[33]
*Magnolia wufengensis* L. Y. Ma et L. R. Wang	The *MAwuPI* gene is only expressed in tepals and stamens and is involved in stamen development in Yulania wufengensis. The ectopic expression of this gene in the Arabidopsis pi-1 mutant can cause the third whorl floral organs to present a filamentous form.	[34]
*Cymbidium faberi* Rolfe	*HoPI* is widely expressed in all floral organs and can restore the stamen and petal development of the Arabidopsis pi-1 mutant, but it cannot restore the development of anthers on the stamens.	[35]
*Lilium longiflorum* Thumb.	The ectopic expression of *LMADS8/9* can rescue the development of the second-round petals in the Arabidopsis pi-1 mutant and transform some sepals into petal-like structures.	[36]
*Phalaenopsis aphrodite* Rchb. f.	The overexpression of the PI-like gene PeMADS6 in *Arabidopsis thaliana* will lead to the transformation of sepals into petals in *Arabidopsis thaliana*.	[37]
*Malus pumila* Mill.	The overexpression of *MdPI* also shows the phenomenon of sepals transforming into petals. In wild-type apples, the length of anthers is similar to that of stigmas, while transgenic apples show that the length of anthers is half of the length of stigmas.	[38]
*Fagopyrum esculentum* Moench	When the buckwheat AP3 gene *FaesAP3* is overexpressed in *Arabidopsis thaliana*, the outer whorl short stamens of the plant become petal-like, and the inner whorl long stamens become filament-like.	[39]
*Medicago truncatula*	Reducing the expression level of *MtNMH7* (*RNAi-MtNMH7*) will lead to slight petal shape defects and stamen carpel-like phenomena in the plant. A decrease in the expression level of MtTomato *MADS6* (*TM6*) will cause some stamens to differentiate into anthers and filaments, but no pollen grains will be produced. When *MtTM6* completely loses its expression, all anthers are completely transformed into carpels.	[40]
*Brassica* L. Plants	The loss of function of the *AP3* gene also shows a trend of stamen-to-carpel transformation.	[41]
*Eriobotrya japonica* (Thunb.) Lindl.	When the *EjAP3* mutant was introduced into *Arabidopsis thaliana*, the transgenic plants showed abnormal traits such as narrower petals and greener stamens.	[42]

**Table 4:** Role of *AG* genes in floral development.

Species	Function Description	Reference
*Rosa* ssp.	Silencing the AG homologous gene *RhAG* in roses and low temperatures can both increase the number of petals. Restricting the expression of *RhAG* in double-petaled roses also results in double-petaled roses.	[45]
*Rosa chinensis*	In double-petaled flowers, the expression level of *RhAG* is lower than that in single-petaled flowers, and the expression domain of *RhAG* in double-petaled flowers shrinks, thereby resulting in a decrease in the number of stamens and an increase in the number of petals in the flower.	[46]
*Cyclamen persicum* Mill.	When the expression of the AG gene *CpAG1* in cyclamen is inhibited, semi-double-petaled flowers with 10 petals appear. When the expressions of *CpAG1/2* are inhibited simultaneously, double-petaled flowers with 40 petals appear.	[47]
*Pisum sativum* L.	After silencing the *PsAGs* genes in peas, the flowers of peas show phenotypes with features such as petalization of stamens and dehiscence of carpels, and an incomplete small flower is also endogenously generated.	[48]
*Chrysanthemum morifolium* Ramat.	By knocking out the *CAG1s* and *CAG2s* genes, it was found that chrysanthemums showed a multi-petal phenotype, and the reproductive organs of both tubular and ligulate flowers were transformed into tubular or ligulate petals.	[49]
*Prunus mume*	*PmAG* in *Prunus mume* is involved in the growth and development processes of multiple vegetative organs. When the *PmAG* gene of *Prunus mume* is overexpressed in *Arabidopsis thaliana*, the petals of the transgenic *Arabidopsis thaliana* plants become smaller, and the stamens and pistils are obviously enlarged.	[28]

**Table 5:** Role of the SEP gene in floral development.

Species	Function Description	Reference
*Triticum aestivum* L.	When the SEP-like gene *TaMADS1* of wheat was transferred into *Arabidopsis thaliana*, *Arabidopsis thaliana* showed the phenomenon of early flowering and also changed the development of floral organs, such as sepals turning into leaves and a reduction in the number of petals and stigmas.	[57]
*Arabidopsis thaliana*	In the *Arabidopsis sep1sep2sep3sep4* quadruple mutant, all four types of floral organs are mutated into leaf-like structures. The *sep1sep2sep3* triple mutant is mutated into sepal-like structures. Except that the expression of *AtSEP3* occurs in the later stage of flower development, *AtSEP1/2/4* are all expressed in the early stage. The *SEP3* mutation will lead to a significant reduction in the number of stamens in the flower, and the stamens are transformed into filamentous carpel structures or fused with carpels.	[58]
*Prunus avium* (L.)	The interaction between *PavSEP* and the *Pav Short Vegetative Phase* (*SVP*) can promote the floral transition.	[59]
*Cucumis sativus* L.	In cucumbers, it has also been found that *SEP* and *SHP* genes interact to regulate the formation of floral organs.	[60]
*Isatis indigotica* Fortune	In *Isatis indigotica*, *IiSEP4* can interact with *IiSVP*, *IiSHP2*, and *IiFruitfull* (*FUL*) to regulate flowering time and the development of stigmas and fruits.	[61]
*Solanum lycopersicum* L.	The inhibition of the *SEP1* homologous gene Tomato *MADS29* (*TM29*) causes the partial transformation of tomato stamens and petals into sepals.	[62]
*Oryza sativa* L.	The SEP gene *OsMADS5/34* can regulate the branching state of rice inflorescences.	[63]
*Prunus mume*	*PmSEP2* and *PmSEP3* are involved in the formation of stamens and pistils in Prunus mume, while *PmSEP4* and *PmSEP1/2* interact pairwise and are involved in the formation of sepals.	[64]

## Data Availability

No new data were created or analyzed in this study. Data sharing is not applicable to this article.
